# Advancing evolutionary medicine in Northern Germany: Collaboration between Kiel University’s Medical Faculty and the Max Planck Institute for Evolutionary Biology

**DOI:** 10.1093/emph/eoae013

**Published:** 2024-07-24

**Authors:** John F Baines, Claudia D Baldus, Frederic Bertels, Monika Brüggemann, Christoph Kaleta, Matthias Laudes, Franz-Josef Mueller, Linda Odenthal-Hesse, Mathilde Poyet, Paul B Rainey, Philip Rosenstiel, Alexander Scheffold, Susanne Sebens, Joachim Thiery, Arne Traulsen

**Affiliations:** Section of Evolutionary Medicine, Institute of Experimental Medicine, Kiel University, Kiel, Germany; Guest Group Evolutionary Medicine, Max Planck Institute for Evolutionary Biology, Plön, Germany; Medical Department II, Hematology and Oncology, University Hospital Schleswig-Holstein, Kiel, Germany; University Cancer Center Schleswig-Holstein (UCCSH), University Hospital Schleswig-Holstein, Kiel, Germany; Department of Microbial Population Biology, Max Planck Institute for Evolutionary Biology, Plön, Germany; Medical Department II, Hematology and Oncology, University Hospital Schleswig-Holstein, Kiel, Germany; University Cancer Center Schleswig-Holstein (UCCSH), University Hospital Schleswig-Holstein, Kiel, Germany; Research Group Medical Systems Biology, Institute of Experimental Medicine, Kiel University, Kiel, Germany; Institute of Diabetes and Clinical Metabolic Research, University Hospital Schleswig-Holstein Campus Kiel, Kiel, Germany; Department of Psychiatry and Psychotherapy, Kiel University and University Hospital Schleswig-Holstein, Kiel, Germany; Department of Genome Regulation, Max Planck Institute for Molecular Genetics, Berlin, Germany; Research Group Meiotic Recombination and Genome Instability, Max Planck Institute for Evolutionary Biology, Plön, Germany; Section of Evolutionary Medicine, Institute of Experimental Medicine, Kiel University, Kiel, Germany; Department of Microbial Population Biology, Max Planck Institute for Evolutionary Biology, Plön, Germany; Laboratory of Biophysics and Evolution, ESPCI, Université Paris Sciences et Lettres, Paris, France; Institute of Clinical Molecular Biology, Kiel University and University Hospital Schleswig-Holstein, Campus Kiel, Kiel, Germany; Institute of Immunology, Kiel University and University Medical Center Schleswig-Holstein, Kiel, Germany; Institute for Experimental Cancer Research, Kiel University, University Hospital Schleswig-Holstein (UKSH), Kiel, Germany; Faculty of Medicine, Kiel University and University Hospital Schleswig-Holstein (UKSH), Kiel, Germany; Department of Theoretical Biology, Max Planck Institute for Evolutionary Biology, Plön, Germany

## INTRODUCTION

In recognition of the significant yet underexplored opportunities that evolutionary medicine presents for advancing biomedical research, clinical practices and public health [1], a symposium entitled, ‘Fostering Interdisciplinary Exchange in Evolutionary Medicine’ was held on 21 November 2023, at the Max Planck Institute for Evolutionary Biology (MPI-EB) in Plön, Germany. The meeting brought together experts from Kiel University’s Medical Faculty and the MPI-EB, with the aim to expand collaboration on multiple levels, including research, teaching and strategic developments.

The potential for synergism between the two institutions in the area of evolutionary medicine was recognized over 15 years ago. This led to the creation of a bridge professorship, a Medical Life Sciences Master Program with a focus area in evolutionary medicine, a graduate training program in applying evolutionary biology to societal challenges (RTG TransEvo) and culminated in a first-of-its-kind Clinician Scientist Program in Evolutionary Medicine (CSEM), which seeks to fill the critical gap in education in evolutionary biology for clinicians during their specialty/residency training.

To sustain and broaden these positive dynamics, a total of 13 scientists and medical professionals presented their latest research findings and perspectives in three sessions covering primary areas where evolutionary insights offer great promise: oncology, inflammation and the host-associated microbiome. Exchange in these fields was then followed by breakout workshops to discuss future developments.

## ONCOLOGY

Evolutionary perspectives are essential in oncology, as they highlight cancer as a process of cellular evolution within the body and help to understand tumor heterogeneity and treatment resistance. Integration of these insights will hopefully lead to the development of treatment strategies that overcome the evolutionary responses of cancer. This session featured presentations that provided an overview of the local research landscape, highlighting theoretical models of cancer evolution, chromatin remodeling in meiotic recombination and research models to study cancer-host evolution in solid tumors.

### Theoretical models of cancer evolution

Mathematical and computational models have a long tradition in cancer biology, where quantitative thinking is critical in guiding research. Haematological malignancies are an area of particular interest in the Kiel Oncology Network (https://www.kon.uni-kiel.de/en), as they allow repeated sampling and subsequent modeling. This is exemplified by the Research Unit ‘Towards a Cure for Adults and Children with Acute Lymphoblastic Leukemia’ (‘CATCH ALL’; https://www.catchall-kfo5010.com). In solid tumors, the accumulation of mutations in an evolving cancer can be heterogenous across space, and carefully designed sampling protocols are needed to ensure that the mutations at the origin of the cancer are correctly identified [2]. Not only genotypic but also phenotypic heterogeneity is important in treating evolving cancers. Optimizing the sequence of different treatments with different selective pressures on an evolving cancer could not only overcome, but even partially exploit this heterogeneity.

### Meiotic epigenetic regulator PRDM9 in cancer

Precision medicine allows personalized and highly specific treatment of diseases, including cancer, but only when reliable biomarkers are available. Cancer exhibits wide variability due to a combination of genetic and epigenetic changes that drive tumorigenesis. A notable example is the PRDM9 protein, which shows variable expression in a significant subset of tumors across various cancer types. Additionally, certain genetic variants of PRDM9 are found more frequently in specific patient populations, such as those who develop B-cell precursor acute lymphoblastic leukemia.

PRDM9 is thus far characterized as a meiotic recombination regulator that places specific epigenetic modifications and interacts with various enzymes to determine the position of double-stranded DNA breaks, initiating homologous recombination, as reviewed in [3]. Risk alleles of PRDM9 are likely underreported because NGS methods fail to capture its coding minisatellite. We thus optimized PRDM9 genotyping and developed tools to analyze its evolution in multiple organisms including mice and humans. This opens up the possibility to investigate PRDM9 allelic diversity as a risk factor in patients with acute lymphoblastic leukemia and other diseases.

### Research models to study cancer-host evolution in solid tumors

Investigating the evolution of solid tumors in the context of their interactions with host/immune cells poses several challenges, as tumors are heterogeneous and dynamic structures, tumor material (especially from metastases) is limited, and liquid biopsies, although easily accessible, only incompletely reflect tumor heterogeneity. Translational experimental research models are therefore crucial for understanding tumor evolution and applying these insights to advance treatments. For this purpose, we use and further develop the following complementary model systems: fixed tumor tissues, stroma-enriched 3D-microtumors (spheroids, organoids), organotypic tissue slices and clinically adapted tumor models.

## INFLAMMATION

Evolutionary perspectives also offer valuable insights into inflammatory disorders, such as trade-offs surrounding certain immune responses and their contribution to susceptibility to modern civilization diseases. The Kiel Region has been a strong focal point for inflammation research since the mid-2000s, significantly propelled by three rounds of funding from the German Excellence Initiative. Both ‘Inflammation at Interfaces’ (2007–2018) and ‘Precision Medicine in Chronic Inflammation’ (2019–2025) included evolutionary components (see, e.g. [4, 5].). Presentations covered diverse topics such as microbiome metabolic activity in inflammatory diseases, T-cell research and epigenetic diagnostics for inflammatory diseases.

### Loss of microbiome metabolic activity in inflammatory diseases

Alterations in gut microbiome composition are consistently observed in inflammatory diseases. However, it is still mostly unclear whether these changes are causative factors or merely secondary effects due to disease. Through constraint-based metabolic modeling, it has been demonstrated that a common feature of microbiome alterations during inflammation is a significant reduction in metabolic functions, including interactions within the microbial community [6]. Notably, in the context of aging, these microbiome changes coincide with the suppression of host processes that rely on microbiome functions and are crucial in the established hallmarks of aging.

### The importance of T-cell research in inflammatory diseases

The evolution of the adaptive immune system in vertebrates is a key step in fine tuning the interactions between hosts and a highly diverse environment, whereby lymphocyte antigen receptor diversity enables precise adaptation to an array of external organisms and antigens, and forms a stable memory. However, this comes at the expense of an increased risk to respond to harmless foreign, e.g. microbiota, or self-antigens. The resulting autoimmune and chronic inflammatory diseases are a central and steadily increasing clinical problem in modern societies. T cells are the key orchestrators of these pathogenic responses. The evolution of a TCR repertoire adapted to both self and environmental antigens, combined with distinct T-cell functional profiles, determines protective and pathogenic immune states. Once established, the longevity of the contributing T cells imprints protective or pathogenic memory. At the Institute of Immunology, Kiel University, several groups study antigen-driven T-cell reactivity against self and environmental antigens to understand how pathogenic immune states are formed, stabilized and potentially therapeutically resolved [7].

### Epigenetic diagnostics for inflammatory diseases

Epigenetics, focusing on inheritable changes in gene expression that do not alter DNA sequences, is revolutionizing cancer diagnosis and shows potential for chronic inflammatory diseases. Technologies such as nanopore sequencing and real-time CpG methylation detection are advancing epigenetic classification at the point of care, enabling accurate intraoperative diagnosis of a wide variety of brain tumors [8]. As epigenetic classification transforms cancer diagnostics by identifying subtypes and predicting treatment responses, chronic inflammatory disorders should be the next focus for this technology. Chronic inflammatory diseases, such as inflammatory bowel disease and rheumatoid arthritis, involve complex interactions of genetic, environmental and epigenetic factors. Through the use of evolutionarily conserved signatures of inflammation, it may be possible to predict therapeutic responses or the likelihood of relapse, thus guiding treatment decisions. This integration aims to enhance personalized medicine by tailoring interventions to individual epigenetic profiles measured at the point of care, thereby improving patient outcomes.

## MICROBIOME

The last two decades have witnessed a paradigm shift in our understanding of the diversity of diseases influenced by the host-associated microbiome. Given the number, diversity and evolvability of members of the microbiome, incorporating eco-evolutionary frameworks are critical to understanding the dynamics between microbial communities and their hosts. In recognition of this importance, microbiome research also became a central research focus at Kiel University, as exemplified by the Collaborative Research Center 1182 ‘Origin and Function of Metaorganisms’, the Kiel Microbiome Center (https://www.uni-kiel.de/en/centres/kmc) to drive translational innovation in microbiome science, and the Global Microbiome Conservancy (GMbC, https://microbiomeconservancy.org/), which is an international initiative to catalyze research capacity at Kiel University, the MPI-EB, and worldwide.

### Contingency genes and the evolution of evolvability

Microbial pathogens and commensals face stringent tests of adaptive potential due to the diversity and polymorphic nature of host immune responses. Persistence often depends on highly mutable sequences that control the expression of immunogenic components, such as polysaccharides, outer membrane proteins and proteinaceous extensins. These sequences, typically short repeats located in promoter regions, mutate at high frequency by slipped-strand mispairing, with changes in repeat copy number affecting binding of RNA polymerase such that genes are turned ON and OFF at high frequency. It has been previously argued that such mutable sequences, so-called ‘contingency loci’, provide bacteria with evolutionary foresight, enabling survival in the face of unpredictable environmental change. A microbe harboring just a handful of contingency genes, each affecting the ON/OFF state of different surface components, can, after a short period of growth, produce phenotypically diverse populations, with the possibility that some variant types not only avoid detection by the host immune response, but proceed to invade host tissues. Experiments are needed to define the conditions under which contingency loci evolve, particularly focusing on the impacts of lineage-level selection [9]. Understanding these selective conditions will help formulate strategies to minimize the risk of microbes, used for therapeutic interventions, evolving harmful traits against hosts.

### Experimental evolution for therapeutic development

Phage therapy is a promising method for the treatment of multidrug-resistant bacterial infections. However, our ability to develop efficacious therapies depends on our understanding of phage biology in order to avoid endless trial-and-error approaches. To that end, it is planned to evolve the already well-studied model phage ΦX174 into a therapeutic agent using experimental evolution approaches. The goals in this process are to increase host range, lower phage resistance rates and understand the impact of life history traits on treatment efficacy.

### Genomic and functional dynamics of human host-microbiome interactions

In parallel with the rise of global urbanization and industrialization, the human gut microbiome drastically shifts both in its composition and diversity. However, the impacts of these changes on host-microbiome interactions remain to be explored. Leveraging the unique resources of the GMbC at Kiel University, we address fundamental questions on the evolution and dynamics of microbiome genomics and functions and their interaction with their host among populations of various levels of urbanization and industrialization. Key genomic and functional characteristics of gut bacteria, including the dynamics of horizontal gene transfer [10] and the degradation of compounds relevant for human health are explored. Studying the relationship between these processes and host lifestyle leads to insight into their potential impact on overall health and susceptibility to disease in an increasingly urbanized world.

## DISCUSSION AND CONCLUSION

After the research presentations, participants engaged in breakout brainstorming sessions aimed at uncovering additional collaboration opportunities. Discussions led to concrete plans for revising and expanding the curriculum of a joint lecture course and seminar series on evolutionary medicine. This initiative is part of a broader commitment to training the next generation of researchers, equipping them with the interdisciplinary insights needed to lead the field forward. It became clear that the intersections across the three research domains were substantial, prompting the organizers to commit to holding exchange symposia twice a year. The symposium concluded with optimism and renewed commitment to future collaboration, with the goal to create Schleswig-Holstein as a hub for evolutionary medicine to drive advancements in understanding and addressing complex medical challenges.
